# Does Anatomic Phenotype of Mitral Annular Disjunction Impact Survival? An Autopsy-Based Retrospective Study

**DOI:** 10.3390/jcdd8120174

**Published:** 2021-12-05

**Authors:** Nan Zhou, Qianhao Zhao, Rui Li, Da Zheng, Yuxi Xiao, Danmi Mao, Yunyi Wang, Jiacheng Yue, Kai Zhang, Jonathan C. Makielski, Jianding Cheng

**Affiliations:** 1 Department of Forensic Pathology, Zhongshan School of Medicine, Sun Yat-sen University, No. 74, Zhongshan 2nd Road, Guangzhou 510080, China; zhoun6@mail2.sysu.edu.cn (N.Z.); zhaoqh5@mail.sysu.edu.cn (Q.Z.); lirui229@mail2.sysu.edu.cn (R.L.); zhengd9@mail.sysu.edu.cn (D.Z.); xiaoyx53@mail2.sysu.edu.cn (Y.X.); maodm@mail2.sysu.edu.cn (D.M.); wangyy285@mail2.sysu.edu.cn (Y.W.); yuejch@mail2.sysu.edu.cn (J.Y.); zhangk47@mail2.sysu.edu.cn (K.Z.); 2 Guangdong Province Translational Forensic Medicine Engineering Technology Research Center, Sun Yat-sen University, Guangzhou 510080, China; 3 Division of Cardiovascular Medicine, Department of Medicine, University of Wisconsin, Madison, WI 53792, USA; jcmakiel@gmail.com

**Keywords:** mitral annular disjunction, prevalence, poor survival, morphology

## Abstract

Controversies have been raised regarding the prevalence and potential clinical significance of mitral annular disjunction (MAD). We aim to address the anatomic characteristics of MAD and their association, if any, on survival. We retrospectively reviewed 1373 consecutive dissected hearts (1017 men, mean age at death 44.9 ± 0.4 y) and frequently detected MAD (median disjunctional length: 2.0 mm, range: 1.5 mm~8.5 mm), with the prevalence of 92.1% over the entire mitral annulus and 74.9% within the posterior annulus (pMAD). The presence of pMAD was associated with increased all-cause mortality (45 y vs. 49 y, hazard ratio [HR]: 1.28, 95% confidence interval [CI]: 1.11~1.47, *p* < 0.001), which persisted in the context of cardiovascular diseases (CVDs; 46 y vs. 51 y, HR: 1.33, 95% CI: 1.14~1.56, *p* < 0.001) but was insignificant in those without CVDs. Compared to those without pMAD, individuals with pMAD affecting the entire posterior annulus or having a mean standardized length of ≥1.78 showed other clinically significant cardiovascular phenotypes, including the enlargement of aortic annular circumferences and a higher occurrence of thoracic aortic aneurysm/dissection. This largest series of autopsies show that MAD is a common phenotype that may exert additive influence on the survival of individuals. It is necessary to establish a precise classification and stratification of MAD.

## 1. Introduction

Mitral annular disjunction (MAD) is characterized by an appreciable separation between the mitral valve-atrial wall junction and the ventricular attachment [1]. MAD has been widely recognized as a prevalent feature of mitral valve prolapse, associated with the severity of valvular pathology, and predicted to cause ventricular arrhythmias [2,3]. Recently, MAD was demonstrated to be independently associated with ventricular arrhythmias, indicating an underrecognized clinical entity predisposed to sudden cardiac death [4,5]. Notably, several independent studies [6,7,8] coincidentally mentioned that MAD appeared associated with younger age at diagnosis. These results motivate additional attention to this special morphological phenotype, MAD, which may have considerable pathophysiological significance. 

A variable incidence of MAD, ranging from approximately 8% to 98%, has previously been reported in living patients, perhaps reflecting discrepant criteria for diagnosis, study population, and/or the imaging modalities utilized in the studies [2,6,7,8,9,10,11,12,13,14]. Using reconstruction of the entire mitral annulus by three-dimensional cardiac computer tomography, Toh et al. [15] recently argued that MAD could be observed in almost all structurally normal hearts, which raised serious doubts about the classical concept of MAD. Is MAD a rare and pathogenic malformation or a common and benign variation? 

Hitherto, most clinical investigations of MAD were based on a relatively small group of living patients, mainly in the context of mitral valvular diseases. Large-scale dissected heart-based studies of MAD have been limited, leaving important questions about the incidence among the general population and the morphological characteristics of MAD. To address these questions, we implemented a large-scale autopsy-based retrospective study to systematically ascertain the morphological characteristics of MAD and determine whether MAD affects individuals’ survival.

## 2. Materials and Methods

### 2.1. Study Population

Consecutive autopsies were collected from the specimen library of Sun Yat-sen University, January 2017 through December 2020. Exclusion criteria were (1) decedents younger than 15 y, (2) decedents who had mitral valve surgeries or heart transplants, and (3) cases where postmortem reports were not available. Personal information (such as gender and age at death), pathological diagnosis, and cause of death were abstracted from postmortem reports. Cases without information of age at death were used to calculate the incidence of MAD but excluded from statistical analysis referring to age. According to the cause of death, cases were classified as a disease- or violence-dominant death. Due to the limited information of clinical records, the diagnosis of a given disease was mainly referred to its macroscopically and microscopically pathological standards. All disease-dominant death were divided into two groups based on the presence or absence of cardiovascular disease (CVDs; mainly including cardiac diseases, major vascular diseases, and cerebrovascular diseases).

### 2.2. Gross Examination

According to the method adopted by Angelini et al. [16], serial sections were prepared by cutting perpendicularly to the mitral annular plane about every 3 mm. MAD was adjudicated when separation from the mitral valvular hinge point to the left ventricular attachment at any section. The disjunctional length was measured from the valvular hinge point to the ventricular attachment. To improve the reliability of the macroscopic adjudication and measurement, we set the cut-off value of ≥1.5 mm. Decedents were then divided into two groups based on the MAD presence (MAD+) or absence (MAD−).

**Figure 1:**
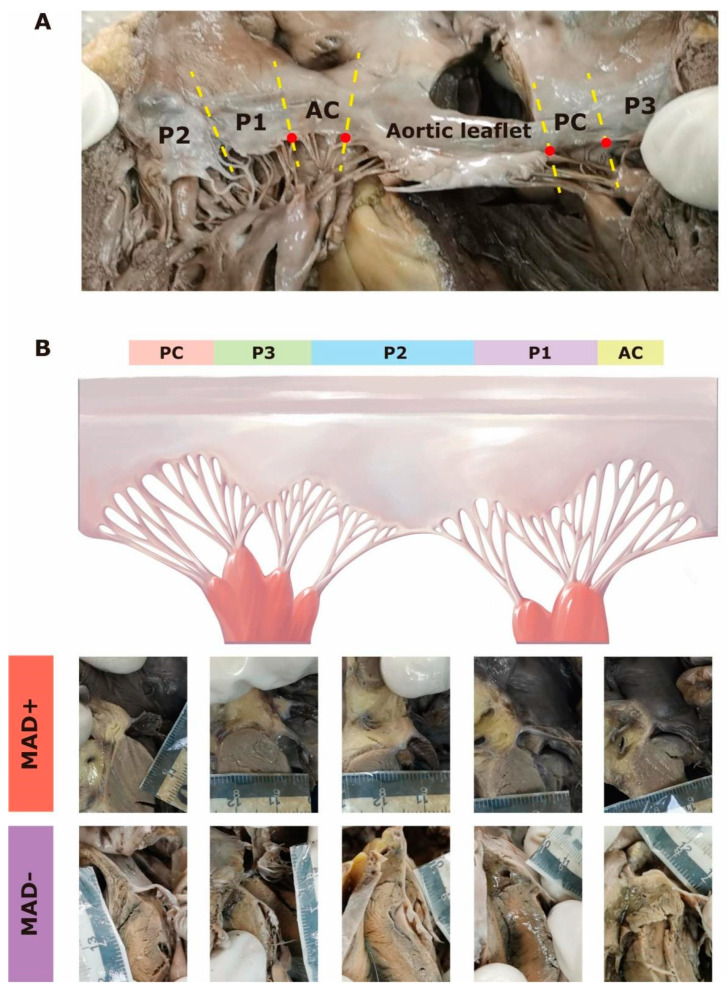
(**A**) Representative picture of the entire mitral annulus (atrial surface, dissecting from the cleft between P2 and P3 region). The mitral annulus was separated into six regions (by yellow dash lines): the anterior region (aortic leaflet), the posterior region (including P1, P2, and P3), and two commissural regions (i.e., AC and PC). Commissural regions were defined as between notches (red dots) of the anterior and the posterior leaflets. (**B**) Schematic diagram of the mitral annulus (upper; atrial surface, dissecting from the middle of the aortic leaflet). Mitral annular disjunction (MAD) could be observed in all regions. Representative pictures of MAD+ and MAD− of each region are shown (lower). AC = anterolateral commissure, PC = posteromedial commissure.

The mitral valve generally comprises two leaflets: the aortic (or anterior) leaflet is in a rounded shape and occupies one-third of the annulus, whereas the mural (or posterior) leaflet is long and narrow that is classified into three regions (P1, P2, and P3) based on the natural clefts, as previously described [17,18]. Connecting the aortic and the mural leaflet are two commissural regions, named the anterolateral and posteromedial commissures [17]. In practice, we defined commissural regions as the relatively small area between the respective notches of the aortic and the mural leaflets (Figure 1A). Thus, the entire mitral annulus was classified into six regions according to its valve attachments, namely the anterior region, the anterolateral commissural (AC) region, and the posterior (P1~P3) region, and the posteromedial commissural (PC) region (Figure 1A).

The maximum disjunctional length of each region was recorded and standardized by the following method. The cut-off value (1.5 mm) was set as the baseline. Regions with a disjunctional length shorter than 1.5 mm, namely MAD− in this study, were adjusted to 0. The standardized length of MAD+ was then calculated as the gross length divided by 1.5 (e.g., gross length of 2.5 mm would be adjusted to 1.67). The longitudinal extent of MAD for each case was expressed as the mean standardized length, calculated as dividing the cumulative standardized length by the number of affected regions (the circumferential extent).

### 2.3. Statistical Analysis

Continuous variables were expressed as the mean ± SEM or median (interquartile range [IQR]) and were analysed using the unpaired Student’s *t*-test or Mann-Whitney test after the tests of normality. Categorical variates were expressed as the count (percentage) and analysed using Fisher’s exact test. Spearman correlation tests were used to interrogate the association between two variates. Survival analyses were performed in the diseases-dominant death group to investigate the association of MAD with long-term outcomes (expressed as the median age at death). Log-rank tests were used to compare the rate of survival free from all-cause mortality between MAD+ and MAD−. Cox proportional hazard regression models were used to analyse the association of MAD with all-cause mortality. Because the age of 35 y is widely used as a cut-off value to define “young” in studies of sudden cardiac death, we compared the survival of individuals with and without MAD in groups <35 y and ≥35 y. Two-tailed *p*-values < 0.05 were considered statistically significant. All statistical analyses were performed on IBM SPSS Statistics 25 (IBM Corp., Armonk, NY, USA).

## 3. Results

### 3.1. Longitudinal and Circumferential Distributions of MAD

A total of 1373 cases (1017 men, mean age at death 44.9 ± 0.4 y) were reviewed and none had mitral valvular degeneration or chordal rupture. The disjunctional length was recorded on 6598 discrete regions. With the cut-off value of ≥1.5 mm, the prevalence of MAD around the entire mitral annulus (circumferential MAD, cMAD) was 92.1% (1264 cases), while it was 74.9% (1028 cases) when only considering the posterior annulus (posterior MAD, pMAD). Along the entire mitral annulus, MAD frequently occurred at two commissural regions (74.3% at PC and 65.9% at AC), while the frequencies of MAD were consistent among different regions of the posterior annulus but appeared higher at P1 (49.4%; Table 1). Except for a smaller group of 223 cases that showed MAD in only one region, MAD was more often detected in more than one region of the mitral annulus, with 14.2% (193 cases) having MAD in all regions. In terms of the posterior annulus, 274 cases (20.0%) had MAD in all three regions (tri-region pMAD), 333 cases (24.3%) had MAD in two regions (bi-region pMAD), and 421 cases (30.1%) had MAD in a single region (mono-region pMAD).

The median disjunctional distance of the mitral annulus was 1.5 mm (range: 0 mm~8.5 mm, IQR: 0 mm~2.5 mm), whereas the median disjunctional length of MAD was 2.0 mm (IQR: 2.0 mm~3.0 mm). The median disjunctional length of MAD varied from different regions, which was longest at the P2 region (2.5 mm [IQR: 2.0 mm~3.0 mm], all *p* < 0.001). After standardization, the median longitudinal extents of cMAD+ and pMAD+ were 1.50 (IQR: 1.33~1.73) and 1.50 (IQR: 1.33~1.78), respectively. Worthy of mention, there was a positive correlation between the longitudinal extent and the circumferential extent of pMAD+ (*r* = 0.29, 95% confidence interval [CI]: 0.23~0.35, *p* < 0.001).

### 3.2. pMAD+ Was Associated with Younger Age at Death

The prevalence of cMAD or pMAD were comparable between men and women (for cMAD: 92.0% in men and 92.1% in women, *p* > 0.99; for pMAD: 75.8% in men and 72.2% in women, *p* = 0.18). There were no significant cardiac morphological differences (including the weight, the thickness of ventricular walls and the circumferences of annuli) between cMAD+ and cMAD− (data not shown), nor were between pMAD+ and pMAD− (Table 2). Histological anomalies, mainly of the heart, were also evaluated, but no association of these microscopic changes was observed with cMAD or pMAD (data not shown). Notably, the mean age at death of pMAD+ was significantly younger than that of pMAD− (43.9 ± 0.5 y vs. 47.8 ± 0.9 y, *p* < 0.001; Table 2), whereas the mean age at death did not differ between cMAD+ and cMAD− (44.8 ± 0.4 y vs. 46.4 ± 1.5 y, *p* = 0.29). 

**Figure 2:**
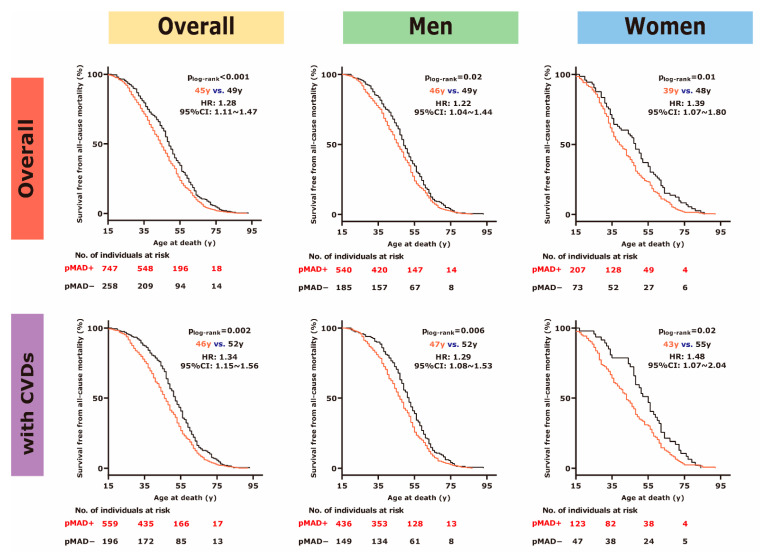
Among 1026 cases of disease-dominant death (21 cases without information of age at death), pMAD+ (red curves) showed poor survival than pMAD− (black curves) with a significant hazard ratio (HR) of all-cause mortality of 1.28 (95% confidence interval [CI]: 1.11~1.47, *p* < 0.001; upper row), which was increased to 1.34 (95% CI: 1.15~1.56, *p* = 0.002) in those with both pMAD and CVDs (lower row; nine cases without information of age at death). CVDs = cardiovascular diseases. pMAD = posterior MAD.

To further analyse the association of long-term survival with the presence of pMAD, we used a group of disease-dominant death (*n* = 1026, 741 men, mean age at death 46.1 ± 0.5 y) because these cases represent a natural endpoint. Compared to pMAD−, the median survival of pMAD+ was decreased by about 4 y (45 y vs. 49 y, *p* < 0.001) with a remarkable hazard ratio (HR) for all-cause mortality of 1.28 (95% CI: 1.11~1.47). The early mortality of pMAD+ existed in those ≥35 y (50 y vs. 52 y, *p* = 0.01) but not in the younger group (28 y vs. 29 y, *p* = 0.49). Women with pMAD showed a 9 y younger age at death than those without (39 y vs. 48 y, HR: 1.39, 95% CI: 1.07~1.80, *p* = 0.01), while pMAD+ men died 3 y earlier than their counterparts (46 y vs. 49 y, HR: 1.22, 95% CI: 1.04~1.44, *p* = 0.02; Figure 2).

Among those with CVDs (*n* = 764, 593 men, mean age at death 48.0 ± 0.5 y), the median survival of pMAD+ remained 5 y younger than pMAD− (46 y vs. 51 y, HR: 1.34, 95% CI: 1.15~1.56, *p* < 0.001), which was also limited to those ≥35 y (51 y vs. 54 y, *p* = 0.008). When concomitant with CVDs, pMAD+ women showed earlier mortality of more than 10 y than their counterparts (43 y vs. 55 y, HR: 1.48, 95% CI: 1.07~2.04, *p* = 0.02), and pMAD+ men had earlier mortality of about 5 y compared to their counterparts (47 y vs. 52 y, HR: 1.29, 95% CI: 1.08~1.53, *p* = 0.006; Figure 2). However, in the context of no CVDs, pMAD+ seemed to have a comparable lifespan to pMAD− (38.5 y vs. 37 y, *p* = 0.95).

### 3.3. Circumferential and Longitudinal Extent Was Associated with Morphological Changes

Association of early mortality with pMAD+ varied among different regions. Compared to pMAD− at a given region, the median survival of pMAD+ was significantly younger at P1 (44 y vs. 48.5 y, *p* < 0.001), which remained younger at P2 (45 y vs. 47 y, *p* < 0.001) but with decreased significances at P3 (45 y vs. 47 y, *p* = 0.33). Additionally, the age at death was correlated with standardized length of P1 (*r* = −0.13, 95% CI: −0.18~−0.07, *p* < 0.001) and P2 (*r* = −0.06, 95% CI: −0.11~−0.01, *p* < 0.001) but not with the standardized length of P3 (*p* = 0.24).

Importantly, the age at death of pMAD+ was negatively correlated with the number of disjunctional regions (*r* = −0.11, 95% CI: −0.16~−0.05, *p* < 0.001), and tri-region pMAD (42.6 ± 0.8 y) appeared to die sooner (*p* = 0.07 for comparing to mono/bi-region pMAD [44.4 ± 0.6 y]; *p* < 0.001 for comparing to pMAD−). Morphologically, tri-region pMAD showed a thinner left ventricular wall and a larger aortic annular circumference than pMAD−, which remained significant after adjusting the weight of hearts (all adjusted *p* < 0.005; Table 3). Compared to pMAD−, a higher occurrence of thoracic aortic aneurysm/dissection (9.1% vs. 3.8%, odds ratio [OR]: 2.56, 95% CI: 1.29~5.11, *p* = 0.007) was observed in tri-region pMAD. No significant morphological change was observed in mono/bi-region pMAD compared to pMAD− (data not shown).

The longitudinal extent of pMAD was also negatively associated with the age at death (*r* = −0.09, 95% CI: −0.14~−0.03, *p* = 0.002). Individuals with upper quarter longitudinal extent (mean standardized length ≥ 1.78; *n* = 279) were three years younger at death than those with lower quarter longitudinal extent (mean standardized length = 0, namely pMAD−; Table 3). Significantly, the upper quarter group had enlarged circumference of all annuli after adjusting the weight of hearts (all *p* < 0.001 for comparing to the lower quarter group, Table 3; all *p* < 0.01 for comparing to the medial quarter group, data not shown). Similarly, a higher incidence of thoracic aortic aneurysm/dissection was observed in the upper quarter group than the lower quarter group (9.3% vs. 3.8%, OR: 2.64, 95% CI: 1.33~5.26, *p* = 0.003). In terms of other morphological parameters, there were no significant differences between the upper and the lower quarter group. None of the significant morphological change was observed in the medial quarter group compared to the lower quarter group (data not shown).

## 4. Discussion

MAD has been attracting increasing attention over the past two decades. Nonetheless, doubts have arisen regarding its pathophysiological importance, and the prevalence and the morphological characteristics of MAD are still in debate [19]. In the current study, we reported an extensive series of dissected hearts for which the entire mitral annulus was carefully evaluated, providing for robust measurement of the incidence of MAD in the general population. Additionally, we found that individuals who had MAD within the posterior annulus suffered worse survival, especially in the context of CVDs, further supporting the pathophysiological importance of MAD. Of crucial importance, we found that the longitudinal and circumferential extent of pMAD is associated with both early mortality and morphological anomalies, shedding light on a potential pathogenic phenotype of MAD.

### 4.1. MAD Is Common along the Entire Mitral Annulus

Although imaging modalities can efficiently diagnose MAD, missed diagnosis of MAD may still happen because much of the annulus is outside the window of detection. Compared to acoustic images, dissected hearts have the advantage that the entire mitral annulus can be evaluated, enabling the intuitive and precise adjudication of MAD. Previous anatomic investigations of MAD were limited in the comprehensiveness of measurements, study population, and the number of hearts evaluated [1,16,20,21].

For the first time, our study comprehensively investigated the entire mitral annulus on the most considerable quantity of dissected hearts and systematically described the longitudinal and circumferential distribution of MAD. Based on 1373 random dissected hearts, our study estimated the incidence of MAD around the entire mitral annulus to be about 92%. More rigorously, we anatomically distinguished the commissural regions from the posterior regions and suggested that MAD was predominant at commissural regions. Our findings further supported that MAD is a common annular variation with a “bimodal distribution” [15,16]. Anatomically, the anterior mitral annulus lies relatively higher than the posterior mitral annulus, making the mitral annulus descend from anterior to posterior and gradually attach to the top of the left ventricle [17,22]. We presumed that this natural feature of the mitral annulus might explain the circumferential distribution of MAD.

Notably, our results show a relatively higher incidence of MAD than previous clinical data [6] from random patients, underlining the value of comprehensive evaluation along the entire mitral annulus when diagnosing MAD. However, it should be noted that anatomic data (“static” hearts) and imaging data (“dynamic” hearts) are not strictly interchangeable. Future research integrating anatomic and imaging data might more precisely define the morphological classification of MAD to shed light on the true prevalence and clinical significance of MAD in the general population.

### 4.2. Pathogenicity of pMAD

Associations between MAD and degenerative valvular diseases, ventricular arrhythmias, and papillary muscular fibrosis have been previously posited, even though only a single long-axis view was evaluated [2,3,6,8,9]. Recently, we reported pilot autopsy-based findings that MAD at the P2 region (≥2.5 mm) was associated with premature cardiac mortality [23]. However, the high prevalence of MAD around the entire mitral annulus has seriously challenged those results.

In the current study, we found that individuals with MAD within the posterior region (pMAD) were relatively younger at death than those without pMAD. These autopsies are not an actual random sample of all deaths, but they were likely to be a reasonable cross-sectional sampling with a diversity of severe underlying health conditions. Thus, these results agree with the overlooked predominance of MAD among young patients in previous cross-sectional studies [6,7,8]. More importantly, based on a group of disease-dominant deaths, we estimated the risk of all-cause mortality increased about 28% in the presence of pMAD, which could ascend to nearly 34% in the context of CVDs. Notwithstanding that disjunctional mitral annulus could prevalently occur in both structurally normal and abnormal hearts [5,6,15], we hypothesized that pMAD might be an anatomic phenotype potentially exerting additive influence on the primary CVDs, accelerating cardiovascular dysfunction and early mortality. Nonetheless, because of potential selection bias in this retrospective group analysis, we cannot ascertain for certain the impact of MAD on the survival of individuals with given CVDs, non-CVDs, or those generally healthy. Prospective studies with more extensive, full-spectrum cohorts and long-term follow-up are needed to confirm or contradict this hypothesis.

### 4.3. Length and Distribution of pMAD Are Predictors for Pathogenicity

While the presence of MAD was acknowledged to be associated with an enlarged mitral annulus [7,8], this study initially identified that pMAD+ in extensive longitudinal or circumferential extent (a less-frequent phenotype) was associated with significant enlargement of aortic annular circumference, suggesting that extensive pMAD might be part of multi-annular malformation [24]. It was unclear whether these anatomic changes influenced valvular dynamics since these enlarged annuli were still within their normal range. Importantly, this study uncovered that extensive pMAD was associated with a higher incidence of thoracic aortic aneurysm/dissection, similar to the prospective outcomes in patients with connective tissue disorders [25,26]. The causality of pMAD, enlarged aortic annulus, and thoracic aortic aneurysm/dissection requires in-depth investigations. We are prone to assume that mechanisms leading to extensive pMAD might also influence the aortic annulus and aortic artery development. Therefore, we propose that MAD presented within the posterior annulus in vast longitudinal or circumferential extent is a rare potential pathogenicity phenotype. Although the current study described a tentative morphological classification of MAD, further investigation focusing on precisely classifying MAD is still of particular interest.

### 4.4. Study Limitations

Although this autopsy-based study included the most significant population to date with a broad spectrum of underlying conditions, it was a retrospective study with inherent limitations to its design. First of all, it was conducted in a single centre only involving limited Chinese decedents with a men-predominance, which limited us to assess MAD in a refining healthy condition. Due to a lack of uniform diagnostic criteria, the condition of MVP in autopsies could hardly be adjudicated in precise. Additionally, our study could not elucidate confounders that might facilitate poor outcomes or interpret the clinicopathological correlation of MAD because of the absent clinical records. Last but not least, we propose a phenotypical classification of MAD and a standardization process assessing the degree of MAD from a limited list of morphological aspects, both of which must be improved. Well-designed investigations that functionally and morphologically evaluate the impact of MAD may be expected to establish a more precise classification and stratification of MAD.

## 5. Conclusions

In summary, MAD is a typical phenotype of the mitral annulus among consecutive Chinese adult autopsies. MAD within the posterior mitral annulus is associated with a remarkably increased risk of all-cause mortality of individuals, especially in the context of CVDs. The longitudinal and circumferential extent of MAD are potential predictors for pathogenicity, motivating the need for a more precise classification of MAD.

## Figures and Tables

**Table 1:** Prevalence of MAD with different cut-off values.

Cut-Off Value	AC ^a^	P1	P2	P3	PC ^a^	cMAD+	pMAD+
≥1.5 mm	824/1251 (65.9)	678/1373 (49.4)	623/1373 (45.4)	608/1373 (44.3)	913/1228 (74.3)	1264/1373 (92.1)	1028/1373 (74.9)
≥2 mm	640/1251 (51.1)	513/1373 (37.3)	536/1373 (39.1)	493/1373 (35.9)	724/1228 (58.9)	1151/1373 (83.8)	897/1373 (65.3)
≥2.5 mm	362/1251 (28.9)	301/1373 (21.9)	359/1373 (26.1)	276/1373 (20.1)	441/1228 (35.9)	843/1373 (61.3)	608/1373 (44.2)
≥3 mm	176/1251 (14.0)	160/1373 (11.6)	207/1373 (15.1)	155/1373 (11.3)	256/1228 (20.1)	558/1373 (40.6)	377/1373 (27.4)
≥4 mm	43/1251 (3.4)	25/1373 (1.8)	60/1373 (4.4)	43/1373 (3.1)	78/1228 (6.3)	181/1373 (13.2)	108/1373 (7.9)

^a ^ The condition of AC in 122 cases and PC in 145 cases was unavailable because those regions were permanently removed during postmortem examination. AC = anterolateral commissural region, PC = posteromedial commissural region, cMAD = circumferential MAD, pMAD = posterior MAD.

**Table 2:** Demography of study population.

	Total (*n* = 1373)	pMAD+ (*n* = 1028)	pMAD− (*n* = 345)	*p*
Age at death (y)	44.9 ± 0.4	43.9 ± 0.5	47.8 ± 0.9	<0.001
Men (%)	1017 (74.1)	771 (75.1)	246 (71.3)	0.18
Height (cm)	164.5 ± 0.2	164.8 ± 0.3	163.4 ± 0.4	0.006
*Anatomy of heart*				
Weight of heart (g)	380.6 ± 2.7	379.0 ± 3.1	385.4 ± 5.4	0.31
Thickness of left ventricular wall (cm)	1.23 ± 0.01	1.22 ± 0.01	1.24 ± 0.01	0.10
Thickness of right ventricular wall (cm)	0.31 ± 0.002	0.31 ± 0.003	0.32 ± 0.004	0.37
Circumference of tricuspid annulus (cm)	11.45 ± 0.02	11.45 ± 0.03	11.43 ± 0.04	0.62
Circumference of pulmonary annulus (cm)	7.82 ± 0.02	7.83 ± 0.03	7.80 ± 0.04	0.60
Circumference of mitral annulus (cm)	9.26 ± 0.03	9.25 ± 0.03	9.26 ± 0.05	0.79
Circumference of aortic annulus (cm)	7.02 ± 0.02	7.03 ± 0.02	7.00 ± 0.04	0.50
*Underlying cardiovascular conditions*				
Coronary atherosclerosis (%)	573 (41.7)	411 (40.0)	162 (47.0)	0.03 ^b^
Thoracic aortic aneurysm/dissection (%)	66 (4.8)	53 (5.2)	13 (3.8)	0.38
Cardiomyopathies (%)	74 (5.4)	57 (5.5)	17 (4.9)	0.78
Otherwise normal heart and vessel (%)	517 (37.7)	395 (38.4)	122 (35.4)	0.34
*Cause of death*				
Diseases-dominant death (%) ^a^	1026 (74.7)	764 (74.5)	262 (75.9)	0.57
Cardiovascular	565 (41.2)	423 (41.2)	142 (41.2)	>0.99
Respiratory	90 (6.6)	81 (7.9)	9 (2.6)	<0.001
Digestive	67 (4.9)	42 (4.1)	25 (7.3)	0.03
Violence-dominant death (%) ^a^	338 (24.6)	257 (25.0)	81 (23.5)	0.61
Trauma	170 (12.4)	122 (11.9)	48 (13.9)	0.34
Poisoning	79 (5.8)	58 (5.6)	21 (6.1)	0.79
Asphyxia	59 (4.3)	50 (4.9)	9 (2.6)	0.09

^a^ The cause of death in 9 decedents was undetermined due to severe body decay; ^b^ *p* = 0.40 after adjusting for the age at death.

**Table 3:** Morphological changes in the extensive longitudinal or circumferential extent of pMAD+.

	pMAD−	Extensive Longitudinal Extent (Tri-Region pMAD)	Extensive Circumferential Extent (Mean Standardized Length > 1.78)
*n*	345	279	274
Weight of heart (g)	385.4 ± 5.4	387.8 ± 6.3	373.0 ± 5.4
Thickness of left ventricular wall (cm)	1.24 ± 0.01	1.24 ± 0.01	1.20 ± 0.01 ^a^
Thickness of right ventricular wall (cm)	0.32 ± 0.004	0.32 ± 0.007	0.30 ± 0.005
Circumference of tricuspid annulus (cm)	11.43 ± 0.04	11.64 ± 0.05 ^b^	11.49 ± 0.05
Circumference of pulmonary annulus (cm)	7.80 ± 0.04	7.98 ± 0.05 ^b^	7.90 ± 0.05
Circumference of mitral annulus (cm)	9.27 ± 0.05	9.46 ± 0.06 ^b^	9.35 ± 0.06
Circumference of aortic annulus (cm)	7.00 ± 0.04	7.19 ± 0.05 ^b^	7.17 ± 0.05 ^a^
Coronary atherosclerosis (%)	162 (47.0)	118 (42.3)	100 (36.5)
Thoracic aortic aneurysm/dissection (%)	13 (3.8)	26 (9.3) ^c^	25 (9.1) ^c^
Cardiomyopathies (%)	17 (4.9)	17 (6.1)	11 (4.0)
Otherwise normal heart and vessel (%)	122 (35.4)	96 (34.4)	109 (39.8)

^a^*p* < 0.01 after adjustment for the weight of heart; ^b^ *p* < 0.001 after adjustment for the weight of heart; ^c^ *p* < 0.01.

## Data Availability

Data sharing is not applicable to this article.

## Sample Availability

Samples of the compounds are not available from the authors.
